# Circulating Tumor DNA and Tissue Testing for Pancreatobiliary Tumors

**DOI:** 10.1001/jamanetworkopen.2025.31373

**Published:** 2025-09-12

**Authors:** Himil Mahadevia, Umair Majeed, Jaydeepbhai Patel, Ahmed K. Ahmed, Ahmed Elhariri, Douaa Albelal, Nakka Naga Malleswara Rao, Hari Krishnareddy Rachamala, Osama Mosalem, Debabrata Mukhopadhyay, Jeremy Jones, Daruka Mahadevan, Mitesh J. Borad, Daniel Ahn, Mohamad Bassam Sonbol, Nguyen Tran, Amit Mahipal, Wen Wee Ma, Robert R. McWilliams, Thor R. Halfdanarson, Lionel A. Kankeu Fonkoua, Tanios Bekaii-Saab, Kabir Mody, Hani Babiker

**Affiliations:** 1 Department of Medicine, University of Missouri–Kansas City; 2 Division of Hematology-Oncology, Department of Medicine, Mayo Clinic Florida, Jacksonville; 3 Department of Biochemistry and Molecular Biology, Mayo Clinic College of Medicine and Sciences, Mayo Clinic Florida, Jacksonville; 4 Division of Hematology-Oncology, Department of Medicine, Mays Cancer Center, UT Health San Antonio, San Antonio, Texas; 5 Division of Hematology-Oncology, Department of Medicine, Mayo Clinic Arizona, Phoenix; 6 Department of Oncology, Mayo Clinic Rochester, Rochester, Minnesota; 7 Division of Hematology-Oncology, Department of Medicine, Case Western Reserve University, Cleveland, Ohio; 8 Division of Hematology-Oncology, Department of Medicine, Cleveland Clinic, Cleveland, Ohio

## Abstract

**Importance:**

The prognosis of advanced pancreaticobiliary tumors is poor. Next-generation sequencing (NGS) of tissue samples is utilized to identify actionable alterations, but there are occasional limitations due to inadequate tissue acquisition. Circulating tumor DNA (ctDNA) is an alternative method, but its correlation with tissue-based NGS remains unexplored.

**Objectives:**

To assess the mutation concordance (mCR) rates between ctDNA and tissue testing for patients with pancreatic ductal adenocarcinoma (PDAC) and cholangiocarcinoma (CC) and to evaluate how ctDNA performs as a treatment response biomarker.

**Design, Setting, and Participants:**

This retrospective cohort study was conducted among patients with PDAC and CC treated at academic institutions from January 2014 to January 2025. Patients underwent ctDNA testing using 1 platform and tissue NGS testing using 2 platforms.

**Main Outcomes and Measures:**

mCR, which measures the shared gene alterations observed by both ctDNA and tissue-based NGS testing, was calculated using the Spearman correlation for PDAC and CC. The performance of ctDNA as a treatment response biomarker was assessed by comparing serial ctDNA data with restaging scans and cancer antigen 19-9 levels in patients with PDAC.

**Results:**

Our cohort included 790 patients: 570 with advanced PDAC (265 [46.5%] female; median [IQR] age, 64 [33-84] years) and 220 with advanced CC (95 [43.2%] female; median [IQR] age, 66 [42-88] years). Overall, 461 patients with PDAC (80.9%) and 192 patients with CC (87.2%) underwent ctDNA testing, while 239 patients with PDAC (41.9%) and 70 patients with CC (31.8%) had tissue NGS testing. Among patients with PDAC, 85 of 130 patients (65.4%) showed shared specific gene alterations between ctDNA and tissue testing; there was a significant mCR, with a Spearman correlation of 0.47 (95% CI, 0.28-0.62; *P* < .001). For patients with CC, 32 of 48 (66.7%) had shared alterations between ctDNA and tissue testing; there was a significant mCR, with a Spearman correlation of 0.56 (95% CI, 0.35-0.70; *P* < .001). A subgroup analysis of patients with PDAC who underwent serial ctDNA testing suggested that new *TP53* subclones and increased ctDNA variant allele frequency levels of *TP53* and *KRAS* were associated with higher odds of progressive disease (eg, increased *TP53 *frequency: odds ratio, 7.28; 95% CI, 2.15-24.66; *P* = .001).

**Conclusions and Relevance:**

In this cohort study of patients with pancreaticobiliary tumors, there was a significant mCR between ctDNA and tissue NGS testing. Additionally, results suggest that ctDNA might detect resistant clones and enable assessment of treatment response.

## Key Points

**Question:**

Does circulating tumor DNA (ctDNA) correlate with next-generation sequencing (NGS) of tissue samples in patients with pancreaticobiliary tumors, and can it help estimate treatment response?

**Findings:**

In this cohort study with 790 patients, ctDNA testing significantly correlated with tissue NGS in finding actionable alterations for patients with pancreatic ductal adenocarcinoma and cholangiocarcinoma. Additionally, serial ctDNA testing found markers associated with higher odds of progressive disease.

**Meaning:**

The findings of this study suggest that ctDNA testing can be used when tissue specimens are insufficient for analysis and can be complementary to tissue-based testing; ctDNA could also act as a marker of response and identify resistance mechanisms.

## Teaser

This cohort study evaluates whether circulating tumor DNA and next-generation sequencing of tissue samples find the same actionable alterations among patients with pancreatic ductal adenocarcinoma and cholangiocarcinoma.

## Introduction

Molecular analysis has become an essential diagnostic component in managing various malignant neoplasms, including pancreaticobiliary (PB) tumors.^1^ The 5-year survival rates of metastatic pancreatic ductal adenocarcinoma (PDAC) and cholangiocarcinoma (CC) are approximately 3.1% and 2%, respectively.^2,3,4^ ADP ribose polymerase inhibitors (PARP inhibitors), which target germline *BRCA1/2* alterations; NTRK (neurotrophic tyrosine receptor kinase) inhibitors, which target various *NTRK* gene fusions; immunotherapy in patients with microsatellite instability-high tumors; and *NRG1* fusions (zenocutuzumab) have shown improved outcomes in patients with PDAC harboring these alterations.^5,6^ Newer targeted drugs, including *FGFR* inhibitors (pemigatinib, infigratinib, and futibatinib), *IDH1* inhibitors (ivosidenib), *BRAF V600E* inhibitors, and *ERBB2 *(formerly known as *HER2/neu*) blockers have also demonstrated clinical benefit in CC after progression on frontline chemotherapy.^7,8,9^

Molecular testing using next-generation sequencing (NGS) on tissue samples obtained by endoscopic ultrasound and fine needle aspiration is used primarily to diagnose and identify targetable alterations in PB malignant neoplasms.^10^ Specimens from endoscopic ultrasound and fine needle aspiration can prove insufficient for complete molecular testing, leading to missed opportunities for targeted therapies. Circulating tumor DNA (ctDNA) testing, or so-called liquid biopsies, holds promise for treatment selection decisions but raises a key question regarding its correlation with tissue analysis. This is critical given that patients may hesitate to undergo additional biopsies and starting treatment promptly is essential.

In addition to treatment selection decisions, optimal therapeutic decision-making requires accurate response assessment. While cancer antigen (CA) 19-9 is the most commonly used biomarker for PB malignant neoplasms, it has limited sensitivity and specificity for monitoring and cannot be used in approximately 15% of patients with PDAC who are CA 19-9 nonsecretors.^11^ It is also less effective when there is discordance between CA 19-9 levels and restaging scans or in cases of mixed response.^12^ Serial ctDNA testing could be used for disease monitoring and identifying the emergence of resistance to treatment.

 We aimed to determine the percentage of shared alterations and the mutation concordance rate (mCR) in PB tumors to assess the reliability of ctDNA testing for treatment decisions. Furthermore, we conducted a subanalysis correlating ctDNA changes with disease progression in imaging and CA 19-9 levels in PDAC secretors and nonsecretors.

## Methods

### Ethics Statement

This retrospective cohort study received institutional review board approval from the Mayo Clinic, and all patient information was deidentified while the data were being collected. Owing to the deidentified data, the need for informed consent was waived. This study was conducted in accordance with the principles outlined in the Declaration of Helsinki, ensuring all participant’s information was deidentified, and the relevant ethics committee approved the research protocol. We followed the Strengthening the Reporting of Observational Studies in Epidemiology (STROBE) reporting guideline for cohort studies.

### Cohort Description

This cohort study was conducted from January 2014 to January 2025 in patients with PB malignant neoplasms seen in phase 1 and PB oncology clinics at the Mayo Clinic (encompassing Arizona, Florida, Minnesota, Iowa, and Wisconsin) and the University of Arizona (Arizona). We included patients (age >18 years) with biopsy-proven advanced PDAC or advanced CC who underwent ctDNA or tissue NGS testing within the institutions. ctDNA, tissue NGS analysis results, and demographic data were obtained from electronic medical records. One ctDNA testing platform and 2 tissue NGS testing platforms, platform 1 and platform 2, were utilized. Eighteen patients who received frontline systemic chemotherapy had serial ctDNA testing. We correlated imaging and CA 19-9 responses with molecular alterations in these patients.

### Testing Platforms

#### ctDNA Blood Testing

This platform sequences more than 73 cancer-associated genes to identify somatic alterations. Cell-free DNA is extracted from plasma, partitioned based on methylation status, enriched for targeted regions, and sequenced using the Illumina platform and hg19 as the reference genome (Guardant360).

#### Tissue Testing

For platform 1 tissue testing, direct sequence analysis was performed on genomic DNA isolated from formalin-fixed paraffin-embedded tumor tissue using the Illumina MiSeq platform, 592 whole gene targets, and in a subsequent test, 700 whole gene targets (Caris). Platform 2 tissue testing utilizes NGS to qualitatively detect substitutions (single nucleotide variants and multi-nucleotide variants) and insertion and deletion alterations in 648 genes (Tempus).

#### Assessment of Actionability

All variants identified as actionable were gathered from ctDNA or tissue testing reports. Those reported as variants of unknown significance were excluded. This study assessed the targetability of each genomic alteration. We characterized an alteration as actionable if it fulfilled the OncoKB level of evidence of 1, 2A, 2B, 3A, 3B, or R1/R2. OncoKB is a precision oncology knowledge base that contains information about the effects and treatment implications of specific cancer gene alterations.

### Statistical Analysis

The mCR was analyzed for patients who underwent NGS of both ctDNA and tissue samples (from either platform 1 or 2 collectively and individually) within a 90-day interval. The ratio of shared alterations between ctDNA and tissue NGS to the total number of alterations in tissue NGS testing as well as ctDNA testing was computed for each patient. Consequently, the percentage of alterations identified in ctDNA testing that were also found in tissue testing as well as the percentage of alterations from tissue testing detected in ctDNA testing were determined for each patient. The mCR was assessed using the Spearman correlation with a significance threshold set at *P* < .05. Due to differences in coverage between the ctDNA and tissue testing, we excluded gene fusions, including *FGFR2* fusions, from concordance analysis. The study was conducted from January 2014 to January 2024 and the analysis continued through January 2025. Using logistic regression, we also correlated imaging and CA 19-9 with molecular responses in patients undergoing multiple ctDNA tests. The threshold for statistical significance in this analysis was *P* < .05. Stata version 15 (StataCorp) was used for the descriptive statistical analysis.

## Results

### Demographic Characteristics

In this study, 790 patients with advanced PB tumors underwent tissue NGS and ctDNA testing between January 2014 and January 2024. Among these, 570 patients were diagnosed with PDAC, while 220 patients had CC. Of the PDAC patients, 265 (46.5%) were female, and 305 (53.5%) were male. Of the CC patients, 95 (43.2%) were female, and 125 (56.8%) were male. The median (IQR) age for PDAC patients was 64 (33-84) years, while the median (IQR) age for CC patients was 66 (42-88) years. All patients with PDAC had either locally advanced or metastatic disease. All patients received at least 1 line of chemotherapy, which was either 5-fluorouracil (5-FU)–based, including 5-FU, leucovorin, irinotecan, and oxaliplatin, or gemcitabine-based, including gemcitabine and nab-paclitaxel as well as gemcitabine monotherapy. All patients with CC had metastatic disease. All these patients received at least 1 line of chemotherapy, including gemcitabine and cisplatin. Overall, 461 patients with PDAC underwent ctDNA and tissue NGS testing on at least 1 of the tissue platforms. Platform 1 and 2 tissue testing were conducted on 135 and 104 patients, respectively. Among platform 1 and 2 tissue testing, most samples (125 of 135 [92.5%] and 94 of 104 [90.3%], respectively) were obtained from the primary pancreas lesion. Table 1 delineates further patient characteristics.

**Table 1.** Characteristics of Patients With PDAC and CC Who Underwent Tissue and/or ctDNA Testing

Characteristic	Patients, No./total No. (%)
**Full sample**	
Sex	
Male	436/790 (55.2)
Female	354790 (44.8)
**Patients with PDAC**	
Sex	
Male	305/570 (53.5)
Female	265/570 (46.5)
Age, median (range), y	64 (38-84)
With ctDNA testing	461/570 (80.9)
With platform 1 tissue testing	135/570 (23.7)
With platform 2 tissue testing	104/570 (18.2)
**Patients with CC**	
Sex	
Male	125/220 (56.8)
Female	95/220 (43.2)
Age, median (range), y	66 (42-88)
With ctDNA testing	192/220 (87.3)
With platform 1 tissue testing	29/220 (13.2)
With platform 2 tissue testing	41/220 (18.6)

Abbreviations: CC, cholangiocarcinoma; ctDNA, circulating tumor DNA; PDAC, pancreatic ductal adenocarcinoma.

### ctDNA and Tissue Correlation

**Figure 1.**
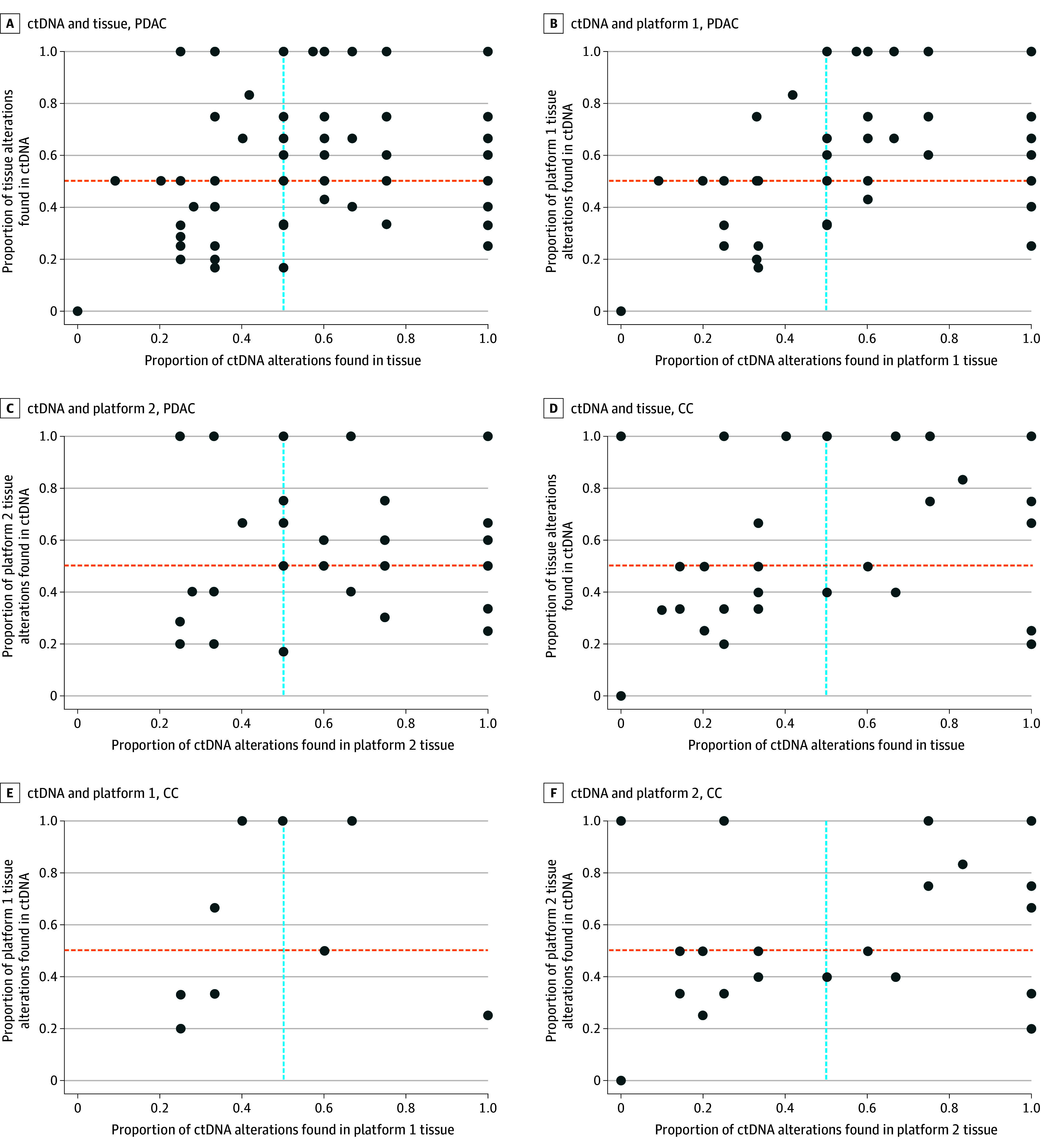
Mutation Concordance Rate Between Tissue and Circulating Tumor DNA (ctDNA) Using Spearman Correlation Each dot represents the correlation for a patient, and the lines indicate the 50% mark. CC indicates cholangiocarcinoma; PDAC, pancreatic ductal adenocarcinoma.

Among 130 patients with PDAC who underwent ctDNA and tissue testing (platform 1 or platform 2) within 90 days of each other, 85 (65.4%) had at least 1 shared specific gene alteration. There was a positive correlation between ctDNA and tissue testing (platform 1 and platform 2) among all patients with PDAC, with a Spearman correlation coefficient of 0.47, which was statistically significant (95% CI, 0.28-0.62; *P* < .001) (Figure 1A).

Among 48 patients with CC who underwent ctDNA and tissue testing (platform 1 or platform 2), 32 (66.7%) exhibited at least 1 shared specific gene alteration. There was a positive correlation between ctDNA and tissue testing (platform 1 and platform 2) among all patients with CC, with a Spearman correlation coefficient of 0.56, which was statistically significant (95% CI, 0.35-0.70; *P* < .001) (Figure 1D).

### ctDNA and Platform 1 Tissue Correlation

**Figure 2.**
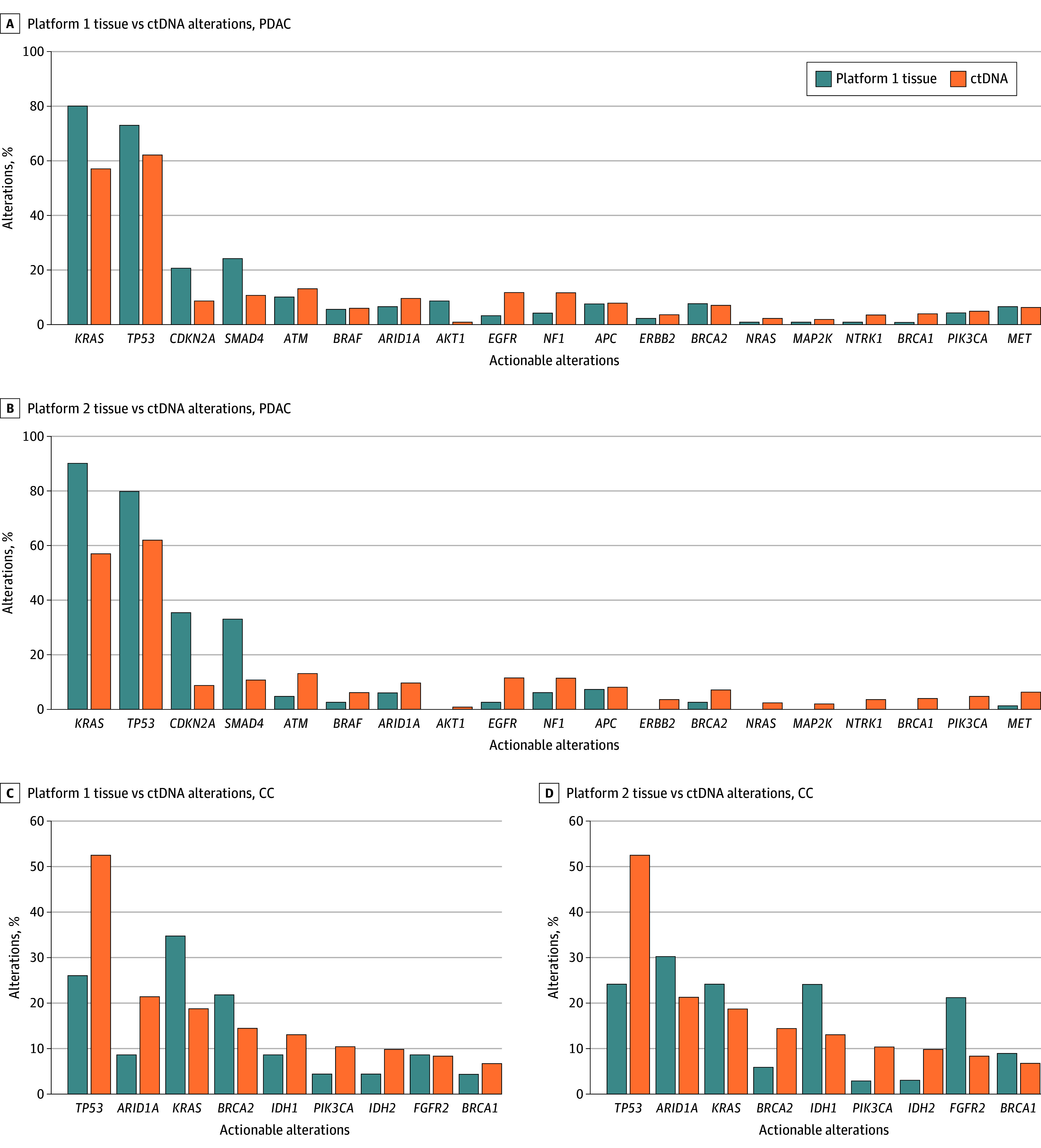
Comparison of Alterations Observed Between Tissue and Circulating Tumor DNA (ctDNA) Testing CC indicates cholangiocarcinoma; PDAC, pancreatic ductal adenocarcinoma.

Among the 77 patients with PDAC who underwent both ctDNA and platform 1 tissue NGS testing (within 90 days of one another), 55 (71.4%) had at least 1 shared specific actionable alteration. There was a positive correlation between ctDNA and platform 1 tissue testing among all patients with PDAC, with a Spearman correlation coefficient for the mCR of 0.58, which was statistically significant (95% CI, 0.38-0.73; *P* < .001) (Figure 1B). Overall, 43 of 77 patients (55.8%) shared *KRAS* alterations between ctDNA and platform 1 tissue testing, whereas 41 patients (53.2%) had shared *TP53* alterations. Figure 2A compares the percentage of different alterations between ctDNA and platform 1 tissue testing for PDAC.

Of the 18 patients with CC who underwent ctDNA blood testing and platform 1 tissue NGS testing, 12 (66.7%) had shared specific gene alterations. There was a positive correlation between ctDNA and platform 1 tissue testing among all patients with CC, with a Spearman correlation coefficient for the mCR of 0.37, but it was not statistically significant (95% CI, −0.27 to 0.78; *P* = .22) (Figure 1E). Figure 2C compares the percentage of different alterations between ctDNA and platform 1 tissue testing for CC.

### ctDNA and Platform 2 Tissue Correlation

Among 53 patients with PDAC who had ctDNA and platform 2 tissue NGS testing (within 90 days of one another), 30 (56.6%) had at least 1 shared specific gene actionable alteration between ctDNA and platform 2 tissue testing. There was a positive correlation between ctDNA and platform 2 tissue testing among all patients with PDAC, with a Spearman correlation coefficient for the mCR of 0.16, which was not statistically significant (95% CI, −0.22 to 0.50; *P* = .39) (Figure 1C). Overall, 25 of 53 patients (47.2%) shared *KRAS* alterations between ctDNA and platform 2 tissue testing, whereas 22 patients (41.5%) had shared *TP53* alterations. Figure 2B compares the percentage of different alterations between ctDNA and platform 2 tissue testing for PDAC

Among 30 patients with CC who underwent ctDNA and platform 2 tissue NGS testing, shared specific gene actionable alterations were found in 20 patients (66.7%). There was a positive correlation between ctDNA and platform 2 tissue testing among all patients with CC, with a Spearman correlation coefficient for the mCR of 0.58, which was statistically significant (95% CI, 0.26-0.79; *P* < .001) (Figure 1F). Figure 2D compares the percentage of different mutations between ctDNA and platform 2 tissue testing for CC.

### Tissue Sample Adequacy for NGS Testing

Overall, 43 of 135 patients with PDAC (31.9%) platform 1 tissue testing had insufficient tissue for testing. Among 104 patients with PDAC who underwent platform 2 tissue testing, samples of 22 patients (21.2%) were inadequate for NGS analysis. Six of 29 patients with CC (20.7%) with platform 1 tissue testing had insufficient tissue for testing, and 8 of 41 (19.5%) with platform 2 tissue testing had insufficient specimens for NGS analysis. All patients with ctDNA blood testing in PDAC and CC had adequate samples for analysis.

### Genomic Alteration Landscape in ctDNA and Tissue NGS Testing

The most common gene altered among patients with PDAC who underwent ctDNA testing was *TP53* (284 of 461 [61.6%]), followed by *KRAS* (262 [56.8%]). The most common alteration we found among *KRAS* alterations was *KRAS G12D* (226 [49.0%]). Other KRAS alterations included *KRAS G12V* (106 [23.0%]), *KRAS G12R* (60 [13.0%]), *KRAS Q61H* (32 [7.0%]) and *KRAS G12C* (7 [1.5%]).

The most common genes altered in platform 1 tissue testing among patients with PDAC were *KRAS* (74 of 92 [80.4%]) and *TP53* (67 [72.8%]). The most common alteration among *KRAS* alterations was *KRAS G12D* (29 [31.5%]). Other *KRAS* alterations include *KRAS G12V* (21 [22.8%]), *KRAS G12R* (9 [9.7%]) and *KRAS Q61H* (6 [6.5%]). We found no patients with *KRAS G12C*. The most common gene altered in platform 2 tissue testing among patients with PDAC was *KRAS* (74 of 82 [90.2%]), followed by *TP53* (66 [80.5%]). The most common specific alteration among all *KRAS* alterations was *KRAS G12D* (32 [39.0%]). Other KRAS alterations included *KRAS G12V* (19 [23.1%]), *KRAS G12R* (17 [20.7%]), *KRAS Q61H* (5 [6.1%]) and *KRAS G12C* (2 [2.4%]). Other commonly altered genes are described in Table 2.

**Table 2.** Targetable Alterations in Tissue Testing Platforms and ctDNA for Pancreatic Adenocarcinoma

Alteration	Patients, No. (%)		
	Tissue platform		ctDNA (blood) (n = 461)
	1 (n = 92)	2 (n = 82)	
*KRAS*	74 (80.4)	74 (90.2)	262 (56.8)
*TP53*	67 (72.8)	66 (80.4)	284 (61.6)
*CDKN2A*	19 (20.6)	29 (35.3)	39 (8.5)
*SMAD4*	22 (23.9)	27 (32.9)	49 (10.6)
*ATM*	9 (9.7)	4 (4.8)	61 (13.2)
*BRAF*	5 (5.4)	2 (2.4)	28 (6.07)
*ARID1A*	6 (6.5)	5 (6.1)	44 (9.5)
*AKT1*	8 (8.6)	0	4 (0.8)
*EGFR*	3 (3.2)	2 (2.4)	53 (11.5)
*NF1*	4 (4.3)	5 (6.1)	53 (11.5)
*APC*	7 (7.6)	6 (7.3)	37 (8.0)
*ERBB2*	2 (2.1)	0	16 (3.4)
*BRCA2*	7 (7.6)	2 (2.4)	33 (7.1)
*NRAS*	1 (1.1)	0	10 (2.1)
*MAP2K*	1 (1.1)	0	9 (1.9)
*NTRK1*	1 (1.1)	0	16 (3.4)
*BRCA1*	1 (1.1)	0	18 (3.9)
*PIK3CA*	4 (4.3)	0	22 (4.7)
*MET*	6 (6.5)	1 (1.2)	29 (6.2)

Abbreviation: ctDNA, circulating DNA.

Among patients with CC, the most common gene altered in ctDNA testing was *TP53* (101 of 192 [52.6%]), followed by *KRAS* (36 [18.8%]). The most common altered gene among patients with CC in platform 1 tissue testing was *KRAS* (8 of 23 [34.8%]). Furthermore, the most common altered gene in platform 2 tissue testing was *ARID1A* (10 of 33 [30.3%]). Other commonly altered genes are described in Table 3.

**Table 3.** Targetable Alterations in Tissue Testing Platforms and ctDNA for Cholangiocarcinoma

Alteration	Patients, No. (%)		
	Tissue platform		ctDNA (blood) (n = 192)
	1 (n = 23)	2 (n = 33)	
*TP53*	6 (26.1)	8 (24.2)	101 (52.6)
*ARID1A*	2 (8.6)	10 (30.3)	41 (21.3)
*KRAS*	8 (34.7)	8 (24.2)	36 (18.8)
*BRCA2*	5 (21.7)	2 (6.06)	28 (14.5)
*IDH1*	2 (8.6)	8 (24.2)	25 (13.0)
*PIK3CA*	1 (4.3)	1 (3.03)	20 (10.4)
*IDH2*	1 (4.3)	1 (3.03)	19 (9.8)
*FGFR2*	2 (8.6)	7 (21.2)	16 (8.3)
*BRCA1*	1 (4.3)	3 (9.09)	13 (6.7)

Abbreviation: ctDNA, circulating DNA.

### Subanalysis of Patients With Serial ctDNA Testing

Eighteen patients with metastatic PDAC who received systemic chemotherapy underwent serial ctDNA testing. Patients received either 5-FU, leucovorin, irinotecan, and oxaliplatin or gemcitabine and nab-paclitaxel. Half the patients were female, and 10 patients (55.6%) were CA 19-9 secretors. *TP53* and *KRAS* alterations were the most common alterations in both CA-19-9 secretors (9 of 10 [90.0%] and 6 of 10 [60.0%], respectively) and nonsecretors (6 of 8 [75.0%] and 4 of 8 [50.0%], respectively). *NF1*, *MET*, *ARID1A*, and *KIT* alterations were only found in nonsecretor patients. ctDNA alterations were associated with CA 19-9 levels and imaging responses using logistic regression. ctDNA levels were measured every 2 months. Disease progression was based on the RECIST 1.1 criteria. There were increased odds of progression on imaging with increasing ctDNA levels of *TP53* with an odds ratio (OR) of 7.28 (95% CI, 2.15-24.66, *P* = .001) and with the appearance of new *TP53* subclones (ie, TP53 R248W, TP53 V272M, and TP53 R273H) with an OR of 5.00 (95% CI, 1.01-24.73; *P* = .04) in all patients. The OR for progression on imaging with increasing ctDNA levels of *KRAS *was 9.33, but the finding was not statistically significant (95% CI, 0.95-91.72; *P* = .06). All patients had progressive disease by the time of their last follow-up. Baseline ctDNA level changes in *TP53* and *KRAS* may be a biomarker of response in PDAC, specifically in nonsecretor patients. *TP53* subclonal mutations were the most common resistant alterations at progression and should be explored as future targets. CA 19-9 levels were not associated with ctDNA levels with an OR of 3.00 (95% CI 0.42-21.04; *P* = *.26*).

## Discussion

PDAC and CC malignant neoplasms have a poor prognosis, especially at an advanced stage.^3,4^ Current chemotherapeutic regimens for PDAC and CC modestly improve progression-free survival and overall survival.^13,14,15,16,17^ Molecular NGS testing is frequently attempted on tissue samples obtained by endoscopic ultrasound and fine needle aspiration in PDAC and CC to find targetable and actionable alterations.^18,19^ Furthermore, the tissue samples are sometimes inadequate for molecular testing as attaining at least 3 cores, cellularity greater than 10% in core needle biopsy tissue NGS, and more than 5 unstained slides for formalin-fixed paraffin-embedded biopsy can be difficult.^20^ In our study, 21% to 32% of tissue samples in PDAC and 20% to 21% of tissue samples in CC were inadequate for testing. Additionally, tissue-based NGS only provides insight into the molecular makeup of the biopsied specimen and thus lacks data on tumor heterogeneity.^21^ ctDNA testing in the blood is accessible via less invasive methods, can better enable us to understand tumor heterogeneity, and allows us to monitor the molecular evolution of the cancer by serial testing.^22,23^

Previous studies have demonstrated the feasibility of ctDNA testing in PDAC and CC.^24,25^ Our study compared ctDNA and tissue NGS testing from 3 vendors for PDAC and CC. We found a positive and significant correlation in targetable alterations between ctDNA and tissue testing for patients with PDAC and CC. Although the correlation coefficients of mCR between ctDNA and tissue NGS testing were modest, with wide confidence intervals, these results suggest that patients with PDAC and CC can rely on ctDNA testing in conjunction with tissue NGS, particularly when tissue specimens are inadequate or not feasible. The correlation was not significant for patients with PDAC whose tissue was tested on platform 2 or for patients with CC on platform 1, likely due to a smaller sample size. The high concordance between the platforms is in line with other studies.^10^ A study^26^ that looked at 145 patients who had tissue and ctDNA testing done found that *TP53* alterations accounted for a significant proportion in both tissue and ctDNA platforms. Another study^27^ that looked at 203 patients with advanced cancers who were enrolled in a phase 1 trial and were profiled by NGS testing found that *TP53* appeared to be a key driver of acquired drug resistance.

*KRAS* was the most altered gene in this study and is now an actionable alteration for most patients with PDAC through clinical trials.^28^ Our study demonstrates that tissue NGS can identify slightly more *KRAS* alterations than ctDNA; this can be related to the level of tumor burden and variability in the shedding of the cancer cells. ctDNA testing also identified *KRAS G12C* in 1.5% of patients with PDAC. A study^29^ that examined the dynamics of altered *KRAS* ctDNA in patients with PDAC undergoing chemotherapy found it to be a highly specific and effective tool for predicting early responses and monitoring treatment. Sotorasib is a small molecular covalent inhibitor that specifically and irreversibly inhibits *KRAS G12C* through a unique interaction with the P2 pocket. It has shown promising clinical activity in patients with solid tumors harboring *KRAS G12C* alteration.^30^ The KRYSTAL-1 study^31^ showed that adagrasib had encouraging clinical activity in pretreated patients with PDAC, CC, and other solid tumors harboring a *KRAS G12C* alteration. A novel noncovalent *KRAS G12D* inhibitor has shown significant preclinical antitumor activity in *KRAS G12D* tumor cells, particularly PDAC.^32^ ctDNA testing can also identify other targetable alterations against which there are US Food and Drug Administration–approved drugs, such as *BRCA1/BRCA2*, *BRAF*, *RET*, *ROS1*, *ERBB2*, *NTRK* alterations, and *NRG1* fusions, albeit rare. Homologous recombination DNA damage repair defects can occur due to alterations in many germline genes, including *BRCA1/2*.^33^ PARP inhibitors are well tolerated and demonstrate sensitivity in PDAC, which has *BRCA1* and *BRCA2* alterations.^34^ *NTRK* fusion inhibitors, such as entrectinib and larotrectinib, have been shown to effectively control the disease in patients with PDAC who are harboring these alterations.^35,36^

*IDH1* alterations are found in a subset of patients with CC. Patients treated with the *IDH1* inhibitor ivosidenib had a PFS of 2.7 months compared with 1.4 months for patients received placebo.^7^ Our study found that *IDH* gene alterations (*IDH1* and *IDH2*) were regularly detected (*IDH1*, 13%; *IDH2*, 9.8%) in ctDNA testing. Thus, ctDNA testing may be reliable in guiding IDH-targeted therapy as an adjunct to tissue NGS testing.

Serial ctDNA testing can reveal tumor evolution and detect treatment resistance early by monitoring changes in *TP53* gene levels and identifying subclones in patients receiving chemotherapy. *TP53* alterations TP53 R273H and TP53 R248W enhance aggressiveness and chemoresistance in PDAC.^37^ Thus, ctDNA testing may help us to identify progression before it is visible clinically or in imaging studies.

### Limitations

This study has limitations, including its retrospective nature, which may introduce selection bias for patients who were assessed for mCR between ctDNA and tissue testing, the need for more data on detailed baseline and prognostic characteristics of patients with PDAC and CC, and the low number of patients with CC, given its rarity. However, our study does provide insight into the role of ctDNA in PB malignant neoplasms. Future studies may stratify patients based on demographic and other baseline variables as well as treatment characteristics and compare mCR between ctDNA and tissue NGS testing.

## Conclusions

In this cohort study of patients with PB tumors, ctDNA identified actionable alterations in patients with PDAC and CC and could be helpful for disease monitoring. This would be especially significant with the advent of novel targeted drugs for PB tumors. Although we acknowledge the relatively modest correlation between ctDNA and tissue NGS testing, ctDNA would offer a noninvasive and, thus, convenient option for patients, particularly those with insufficient tissue samples for NGS testing.

## Notes

Supplement.
Data Sharing Statement
