# Supplementary material for: Circulating Tumor DNA and Tissue Testing for Pancreatobiliary Tumors
Source: JAMA Netw Open. 2025 Sep 12;8(9):e2531373. doi: 10.1001/jamanetworkopen.2025.31373 (PMC12432637; doi:10.1001/jamanetworkopen.2025.31373)
Supplement: Supplement. — Data Sharing Statement [file jamanetwopen-e2531373-s001.pdf]

Data Sharing Statement

Mahadevia. Circulating Tumor DNA and Tissue Testing for Pancreatobiliary Tumors. *JAMA Netw Open*. Published September 12, 2025. doi:10.1001/jamanetworkopen.2025.31373

Data
Data available: No
